# RETRACTION: Overexpression of hsa_circ_0061817 Can Inhibit the Proliferation and Invasion of Lung Cancer Cells Based on Active Compounds

**DOI:** 10.1155/omcl/9832815

**Published:** 2025-09-09

**Authors:** Oxidative Medicine and Cellular Longevity

RETRACTION: L. Ye, Y. Zhong, L. Hu, et al., “Overexpression of hsa_circ_0061817 Can Inhibit the Proliferation and Invasion of Lung Cancer Cells Based on Active Compounds,” *Oxidative Medicine and Cellular Longevity* 2022 (2022): 4509019, https://doi.org/10.1155/2022/4509019.

The above article, published online on 16 September 2022 in Wiley Online Library (wileyonlinelibrary.com), has been retracted by John Wiley & Sons Ltd.

This article has been retracted following an investigation undertaken by the publisher. This investigation has uncovered evidence of one or more of the following indicators of systematic manipulation of the publication process:

1. Discrepancies in scope
2. Discrepancies in the description of the research reported
3. Discrepancies between the availability of data and the research described
4. Inappropriate citations
5. Incoherent, meaningless and/or irrelevant content included in the article
6. Manipulated or compromised peer review

The presence of these indicators undermines our confidence in the integrity of the article's content and we cannot, therefore, vouch for its reliability. Please note that this notice is intended solely to alert readers that the content of this article is unreliable. We have not investigated whether authors were aware of or involved in the systematic manipulation of the publication process.

The authors disagree to the retraction.

